# X;7 Translocation at p21.1 and q31.31 Disrupting *DMD*: A Multiomics Study of a Male Dystrophinopathy Case

**DOI:** 10.1155/humu/8404135

**Published:** 2026-03-23

**Authors:** Rui Dong, Kai Ma, Yuexia Bai, Xiaochen Wang, Min Gao, Hong Qin, Kaihui Zhang, Guohua Liu

**Affiliations:** ^1^ Pediatric Research Institute Children′s Hospital Affiliated to Shandong University (Jinan Children′s Hospital) Jinan Shandong Province China; ^2^ Shandong Provincial Clinical Research Center for Children′s Health and Disease Children′s Hospital Affiliated to Shandong University (Jinan Children′s Hospital) Jinan Shandong Province China; ^3^ Department of Neurology Children′s Hospital Affiliated to Shandong University (Jinan Children′s Hospital) Jinan Shandong Province China; ^4^ Department of Pathology Children′s Hospital Affiliated to Shandong University (Jinan Children′s Hospital) Jinan Shandong Province China; ^5^ Department of General Surgery Children′s Hospital Affiliated to Shandong University (Jinan Children′s Hospital) Jinan Shandong Province China

## Abstract

**Background:**

Dystrophinopathy comprises a group of X‐linked recessive muscular disorders that primarily affect skeletal and/or cardiac muscles. Although more than 90% of dystrophinopathy patients exhibit detectable variants in the *DMD* gene, a small proportion of cases remain undiagnosed at the molecular level.

**Methods:**

We employed an integrated multiomics approach, including RNA sequencing (RNA‐seq) of muscle tissue, optical genome mapping (OGM), short‐ and long‐read whole‐genome sequencing (WGS), and karyotype analysis to elucidate the genetic mechanism in a dystrophinopathy family.

**Results:**

Integrated genomic analyses identified a balanced reciprocal translocation between Xp21.1 and 7q31.31 in the proband, with the X‐chromosomal breakpoint disrupting intron 20 of *DMD*. RNA sequencing of muscle tissue revealed severely reduced *DMD* transcript coverage downstream of the breakpoint, consistent with transcriptional interruption. Muscle immunohistochemistry showed absent dystrophin staining at the N‐terminus, rod domain, and C‐terminus, confirming loss of protein expression. The translocation was maternally inherited; the mother is a carrier with a dystrophinopathy phenotype.

**Conclusions:**

We report a rare male case of dystrophinopathy caused by an X;autosome translocation disrupting *DMD*, thereby expanding the mutational spectrum of this disorder. Our findings underscore the value of comprehensive genomic profiling in resolving diagnostically elusive dystrophinopathy cases.

## 1. Introduction

Dystrophinopathy represents a group of X‐linked recessive muscular disorders, primarily affecting skeletal and/or cardiac muscles due to pathogenic variants in the *dystrophin* (*DMD*) gene located at Xp21 [1]. This group includes Duchenne muscular dystrophy (DMD), Becker muscular dystrophy (BMD), and X‐linked dilated cardiomyopathy (XLDCM), with phenotypes resulting from qualitative and/or quantitative abnormalities in dystrophin isoforms, notably the deficiency of Dp427m leading to classic presentations [2]. Although predominantly affecting males, symptomatic manifestations in female carriers are rare, with only 2.5%–7.8% reporting muscle or cardiac involvement [3–5]. The *DMD* gene′s complex structure leads to a wide array of pathogenic variants, including single nucleotide variants, small insertions/deletions, exon deletions/duplications, and structural variations, with the Human Gene Mutation Database (HGMD) documenting 5855 different *DMD* variants [6]. Such complexity complicates genetic diagnosis in some cases. Accurate molecular genetic diagnosis is crucial for effective clinical management, multidisciplinary care, genetic counseling, prenatal diagnosis, and eligibility for emerging therapies.

In a family with unresolved dystrophinopathy, conventional genetic testing failed to identify a causative variant. To address this diagnostic gap, we applied an integrated multiomics strategy combining muscle RNA sequencing, optical genome mapping (OGM), short‐ and long‐read whole‐genome sequencing (WGS), and karyotyping. We identified a male proband carrying a balanced t(X;7)(p21.1;q31.31) translocation, with the X‐chromosomal breakpoint disrupting intron 20 of *DMD*. This rearrangement leads to transcriptional interruption, complete dystrophin deficiency, and a severe DMD‐like phenotype. The translocation was maternally inherited; his carrier mother manifests a milder, BMD‐like clinical presentation.

## 2. Material and Methods

### 2.1. Ethics Statement

Approval for this study was obtained from the Institutional Review Board of the Children′s Hospital Affiliated to Shandong University (Approval No. SEFE‐IRB/P‐2022015). Written informed consent was obtained from the legal guardians of the minor participants prior to their enrollment in the study.

### 2.2. Patient Recruitment

A family with clinical suspicion of dystrophinopathy was recruited, and detailed medical history, physical examination findings, and laboratory test results were recorded. Peripheral blood samples were collected for all genomic analyses, and a skeletal muscle biopsy was obtained from the proband for RNA studies.

### 2.3. Multiplex Ligation‐Dependent Probe Amplification (MLPA) Analysis

MLPA was performed on 100 ng genomic DNA using SALSA MLPA P034‐B2 DMD‐1 and P035‐B1 DMD‐2 kits (MRC‐Holland). Probes were hybridized, ligated, and PCR‐amplified with fluorescent primers according to the manufacturer′s protocol. Fragments were separated on an ABI 3730xl and analyzed using GeneMapper v4.0; copy number variants (CNVs) were called with Coffalyser.Net (MRC‐Holland).

### 2.4. Trio‐Whole Exome Sequencing (WES) Analysis

WES was performed using the IDT xGen Exome Research Panel v2 for target enrichment and sequenced on an Illumina NovaSeq 6000. Reads were aligned to GRCh37/hg19 using Sentieon. Variants were annotated and interpreted following the American College of Medical Genetics and Genomics (ACMG) guidelines, integrating population databases, in silico predictions, and clinical phenotype.

### 2.5. Muscle Biopsy RNA Sequencing (RNA‐Seq)

RNA‐seq was performed on muscle biopsies preserved in RNAsolid (Servicebio, Wuhan, China). Total RNA was extracted using the RNeasy Mini Kit (QIAGEN, Hilden, Germany) and assessed for integrity. mRNA was enriched with oligo (dT) beads, fragmented, and reverse‐transcribed into cDNA. Libraries were prepared using the Illumina TruSeq RNA Library Prep Kit and sequenced on a NovaSeq 6000. Clean reads were aligned to GRCh38/hg38 using HISAT2, and alternative splicing events were analyzed and visualized in IGV.

### 2.6. RT‐PCR Validation

To validate the splicing abnormality detected by RNA‐seq, RT‐PCR was performed on total RNA from muscle tissue using SuperScript IV Reverse Transcriptase (Thermo Fisher Scientific, Waltham, Massachusettes, United States). PCR amplification employed primers flanking the putative skipped region: DMD‐F1 (Exon 19)—5 ^′^‐CTGCAAGATGCCAGCAGATC‐3 ^′^; and DMD‐R1 (Exon 65)—5 ^′^‐GTTGTGCTCTTGCTCCAGGC‐3 ^′^. Products were resolved on a 2% agarose gel and visualized with the GelDoc imaging system (Bio‐Rad, Hercules, California, United States). *β*‐Actin served as an internal control.

### 2.7. OGM

High–molecular‐weight genomic DNA was extracted using the Prep Blood and Cell Culture DNA Isolation Kit (Bionano Genomics, San Diego, California, United States). DNA labeling was performed with the Prep DLS DNA Labeling Kit (Bionano Genomics, San Diego, California, United States) using direct labeling enzyme DLE‐1 and DLGreen fluorophores, followed by proteinase K digestion and cleanup. Labeled DNA was stained, reduced with dithiothreitol, diluted in flow buffer, and homogenized overnight at 4°C. Samples were loaded onto Saphyr chips and imaged on the Bionano Saphyr system (Bionano Genomics, San Diego, California, United States), achieving a total effective coverage of 137.89×. Quality metrics included N50 = 237.75 kbp, mapping rate = 85.9%, and label density consistent with high‐quality data. *De novo* genome map assembly and structural variant (SV) calling were performed against the GRCh38/hg38 reference using Bionano Access v1.7.1 and Bionano Solve Tools (Bionano Genomics, San Diego, California, United States).

### 2.8. Short‐read WGS

Genomic DNA was fragmented to 150–200 bp using enzymatic digestion and libraries were constructed with the FreySeq Whole Genome Library Preparation Kit (Frey Genetics, Suzhou, China). Sequencing was performed on the DNBSEQ‐T7 platform (MGI Tech, Shenzhen, China) with 150‐bp paired‐end reads. Raw reads in FASTQ format were aligned to GRCh37/hg19 using BWA‐MEM. PCR duplicates were marked and variants were called using Sentieon. Variants were annotated with ANNOVAR and prioritized using in silico predictors. Pathogenicity assessment followed ACMG guidelines, integrating population frequency, computational evidence, and clinical phenotype.

### 2.9. Long‐read WGS

High‐molecular‐weight genomic DNA was isolated. DNA integrity was confirmed by pulsed‐field agarose gel electrophoresis, and concentration was quantified using Nanodrop 2000 (Thermo Scientific, United States) and Qubit 3.0 fluorometer (Life Technologies, United States). Approximately 8 *μ*g of DNA was fragmented using a g‐Tube (Covaris, United States) at 1600 × g, followed by purification and concentration with SMRTbell cleanup beads (PacBio, United States). SMRTbell libraries were prepared using the SMRTbell Prep Kit 3.0 (PacBio, United States) according to the manufacturer′s protocol and sequenced on the PacBio Revio system. Raw data were processed using SMRT Link v25.1 (PacBio) to generate HiFi reads. Circular consensus sequences (CCS) were mapped to the GRCh37/hg19 reference genome using pbmm2 v1.10.0. Single‐nucleotide variants (SNVs) were called with DeepVariant v0.7.0 and annotated using VEP v107. SVs were detected by Sniffles v2.0.7 and cuteSV v1.0.12, then annotated with AnnotSV v3.1.1. Short tandem repeats (STRs) were identified using GrandSTR v1.3.6 (Grandomics).

### 2.10. Chromosome Karyotype Analysis

Peripheral blood lymphocytes were cultured in RPMI 1640 at 37°C for 72 h, arrested in metaphase with colchicine, and subjected to G‐banding for karyotype analysis at a resolution of 320–400 bands per haploid genome.

## 3. Results

### 3.1. Clinical Presentation and Histopathological Findings

The proband is a 6‐year‐old boy, the second child of nonconsanguineous parents. He was delivered by cesarean section at term with neonatal asphyxia. His clinical presentation was consistent with DMD, with symptom onset at age 3. Key signs include bilateral lower limb weakness, frequent falls, waddling gait, inability to run or jump, and difficulty climbing stairs or rising from a squat. Physical examination revealed upper limb strength of 5/5 and lower limb strength of 4/5, hypotonia, calf muscle pseudohypertrophy, and positive Gower′s sign. Cognitive and language development were normal.

Laboratory tests showed elevated serum creatine kinase (CK) levels at 18,915 U/L. Electromyography (EMG) of all limbs showed myopathic changes, and magnetic resonance imaging (MRI) revealed fibro‐fatty replacement in the bilateral thighs and buttocks. Muscle biopsy of the left gastrocnemius showed moderate variability in muscle fiber size, numerous small round fibers, scattered necrotic and regenerating fibers, mild endomysial proliferation, and perimysial fat infiltration. Immunohistochemistry showed absent staining for dystrophin rod domain (dystrophin‐R), C‐terminus (dystrophin‐C), and N‐terminus (dystrophin‐N) (Figure 1).

Figure 1Clinical and diagnostic findings. (a) Clinical appearance of the legs in the proband and his mother, showing signs of calf muscle pseudohypertrophy. (b) Family pedigree illustrating a pattern consistent with dystrophinopathy, where the female carrier exhibits clinical symptoms. (c) MRI results of the proband, indicating muscle involvement and potential disease progression. (d) Muscle biopsy pathological results from the proband, demonstrating characteristic dystrophic changes (100×). Immunohistochemistry panels show negative staining for dystrophin‐R, dystrophin‐C, and dystrophin‐N (100×).

**Figure:**
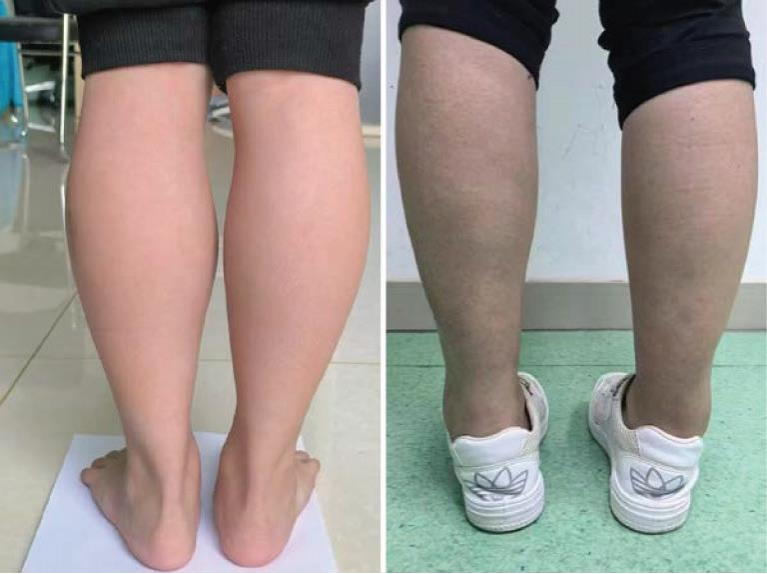
(a)

**Figure:**
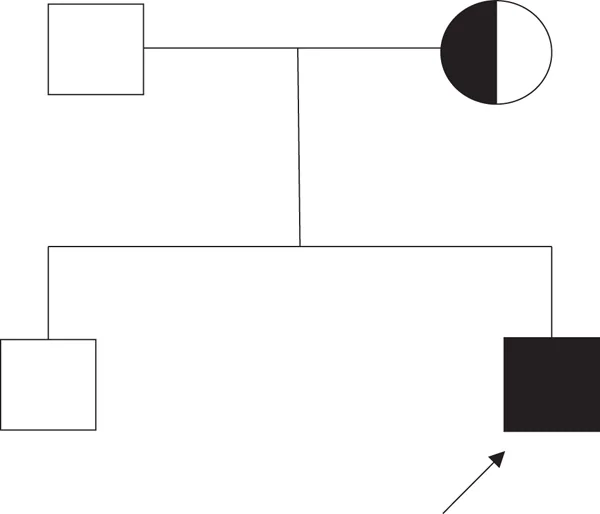
(b)

**Figure:**
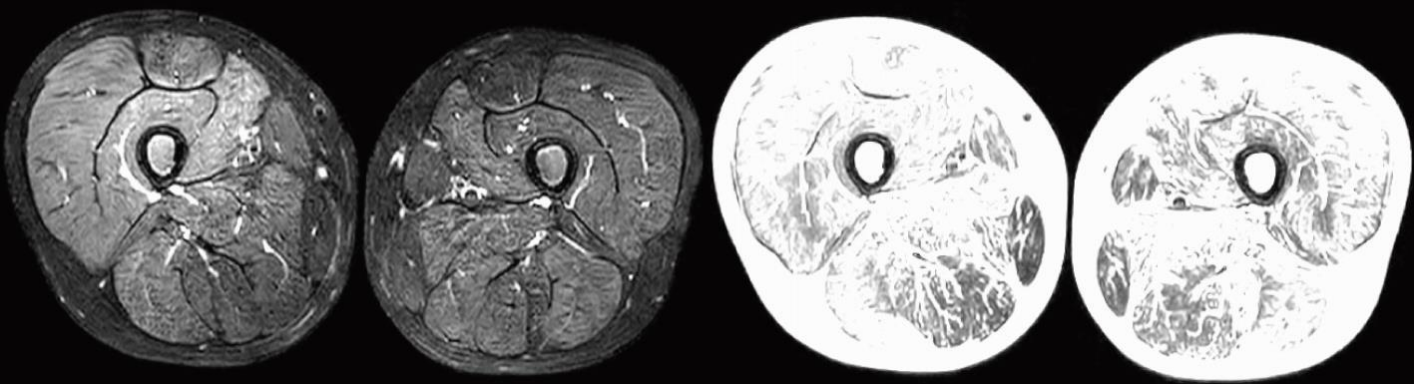
(c)

**Figure:**
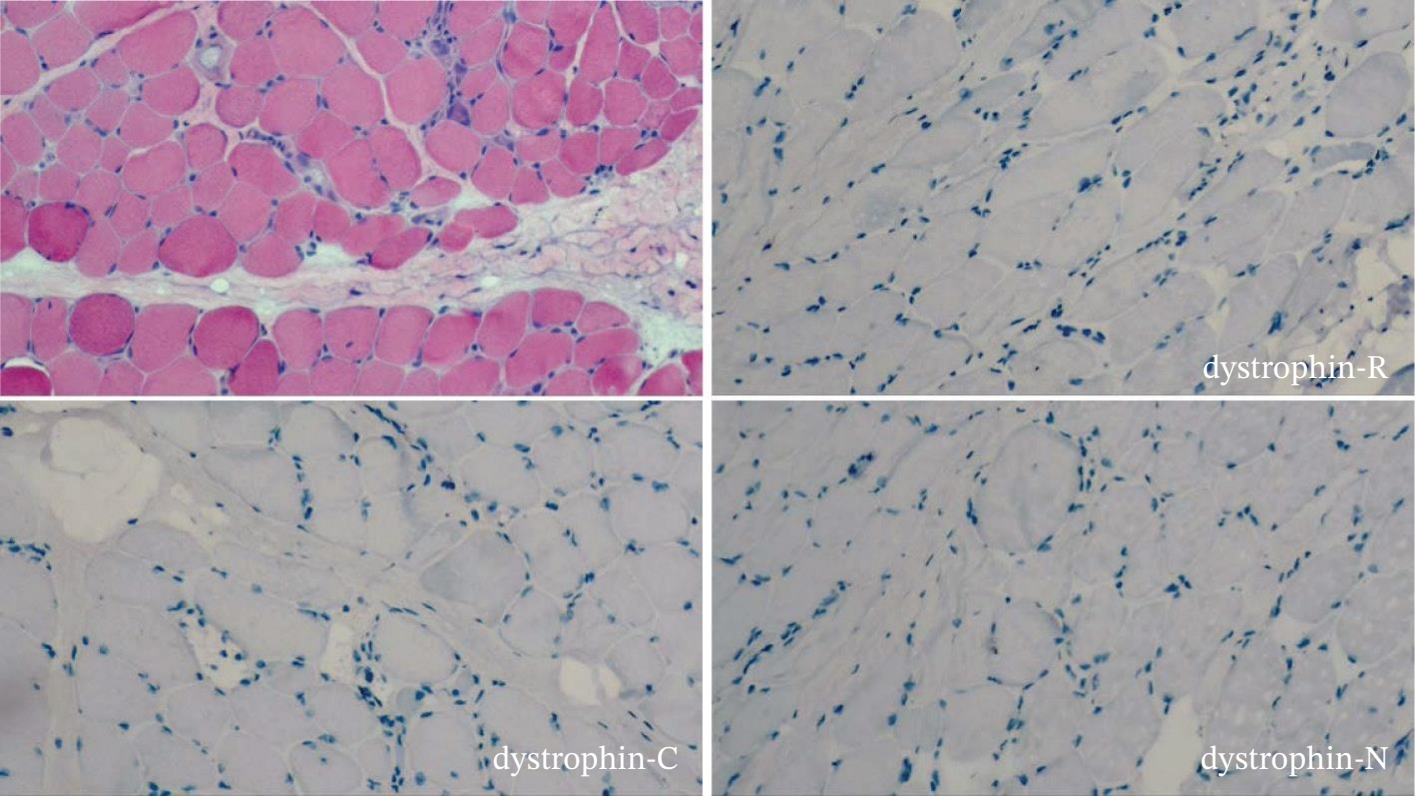
(d)

The mother exhibited early‐onset muscle weakness since childhood, characterized by progressive lower limb weakness, abnormal gait, and mild calf muscle hypertrophy. Her CK level was elevated at 401 U/L. Prior to the birth of her first child, she experienced two natural miscarriages. Notably, neither her elder brother nor her father displayed any signs or symptoms related to muscle disease.

### 3.2. MLPA Analysis of the *DMD* Gene

MLPA analysis of the *DMD* gene (NM_004006.3; Exons 1–79) revealed no exonic copy number changes in the proband.

### 3.3. Trio‐WES Reveals *COL6A3* Variants Unrelated to Phenotype

Trio‐WES identified three variants of uncertain significance (VUS) in the paternal *COL6A3* gene (NM_004369.4): c.4814 T > C (p.Ile1605Thr), c.7224C > G (p.Asp2408Glu), and c.7979A > G (p.Asn2660Ser) (GRCh37/hg19). No point variants or small indels were detected in the *DMD* gene. Considering the proband′s phenotype and family history, these *COL6A3* variants are likely unrelated to the proband′s condition.

### 3.4. Abnormal *DMD* Splicing Detected by RNA‐seq in Muscle Tissue

RNA‐seq of the proband′s muscle tissue revealed severely reduced or absent read coverage across *DMD* Exons 21–62 (Figure 2), with apparent splicing connections between upstream and downstream exons. This pattern could reflect either a large in‐frame exon skipping event or transcriptional interruption due to a SV disrupting the *DMD* locus.

**Figure 2:**
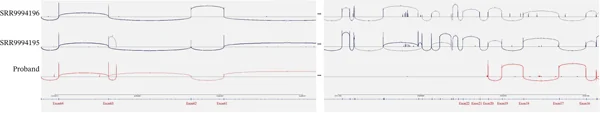
Abnormal *DMD* splicing pattern detected by RNA‐seq in proband muscle tissue. Short‐read RNA‐seq alignment shows severely reduced or absent read coverage across Exons 21–62, with a single apparent splicing junction (indicated by red arc) linking Exon 20 to Exon 63.

### 3.5. Lack of RT‐PCR Confirmation for Predicted *DMD* Splicing Aberration

RT‐PCR using primers in *DMD* Exon 19 and Exon 65 failed to amplify the expected product corresponding to Exons 21–62 skipping (Figure S1). Successful amplification of *β*‐actin confirmed adequate RNA quality and cDNA synthesis. The absence of detectable transcripts is more consistent with transcriptional interruption rather than stable expression of an aberrant isoform.

### 3.6. OGM Identifies a t(X;7) Translocation Involving *DMD*

OGM detected a translocation involving Chromosomes X and 7 in the proband, with breakpoints at chrX:32,477,780–32,490,617 and chr7:118,205,882–118,229,562 (GRCh38). The translocation is speculated to result in two derivative chromosomes, der (7)(7pter →7q31.31::Xp21.1 → Xpter) and der (X)(7qter →7q31.31::Xp21.1 → Xqter), potentially disrupting the *DMD* gene on Chromosome X and involving the *ANKRD7* locus on Chromosome 7. This interval overlaps *DMD* Intron 20 (Figure 3).

Figure 3Schematic of the t(7;X) translocation detected by OGM (GRCh38). (a) The translocation resulted in the formation of two derivative chromosomes: der(7) (7pter →7q31.31::Xp21.1 → Xpter) and der(X) (7qter →7q31.31::Xp21.1 → Xqter). (b) The predicted breakpoints are located at (chr7:118205882, chrX:32477780) and (chr7:118229562, chrX:32490617).

**Figure:**
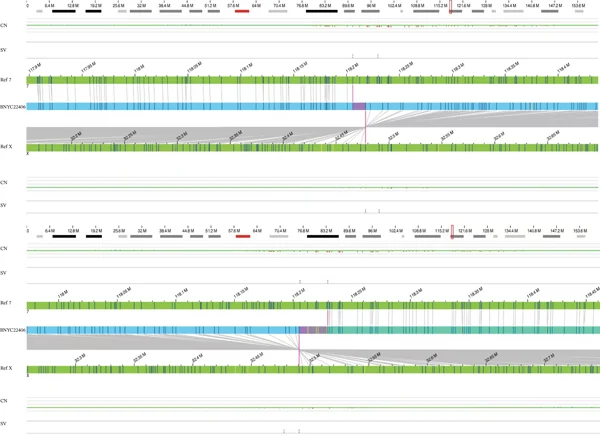
(a)

**Figure:**
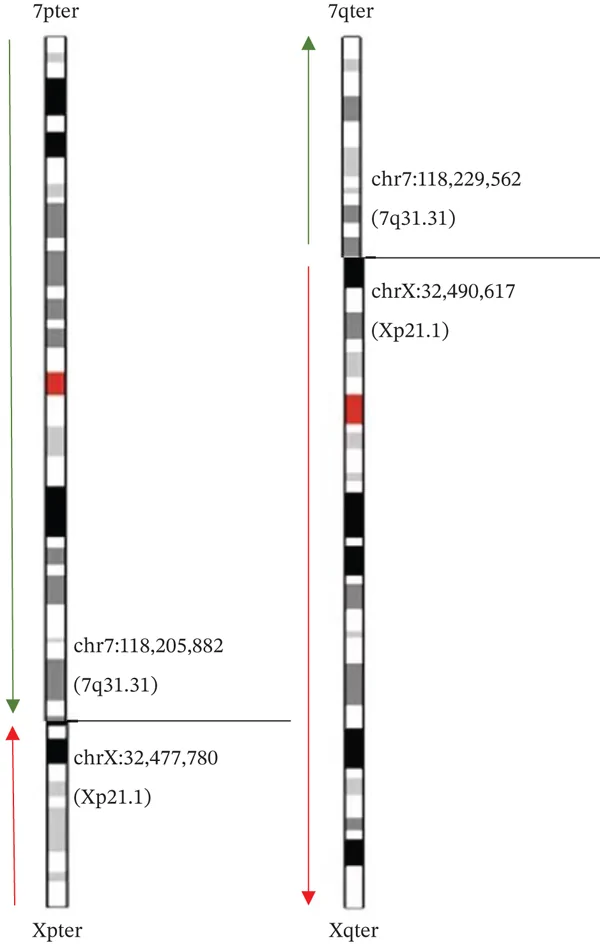
(b)

### 3.7. WGS for SV Detection

Short‐read WGS corroborated the structural rearrangement identified by OGM, revealing a translocation between Chromosomes X and 7 with breakpoints at chrX:32,488,583–32,488,724 and chr7:118,206,930–118,207,094 (GRCh38) (Figure 4). The X‐chromosome breakpoint lies within Intron 20 of the *DMD* gene, consistent with OGM findings. The Chromosome 7 breakpoint is located approximately 7.6 kb upstream of the *ANKRD7* gene, within a TATA repeat‐rich region between *LSM8* and *ANKRD7*.

**Figure 4:**
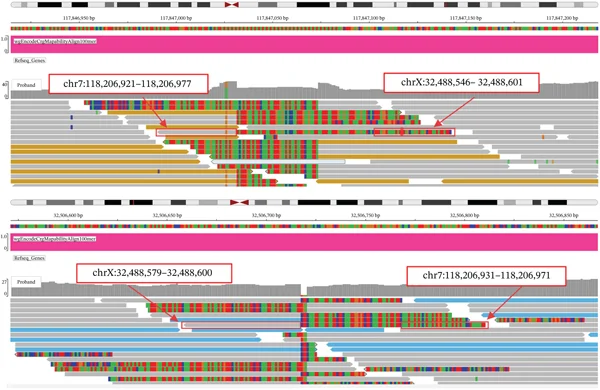
Validation of the t(7; X) translocation breakpoint by WGS (GRCh38). WGS reveals the breakpoint region on Chromosome 7 and X. Coordinates are indicated on the plot.

Long‐read WGS resolved the t(X;7) breakpoints at single‐nucleotide resolution, mapping them to chrX:32,488,588 and chr7:118,207,020 (GRCh38)—corresponding to Intron 20 of *DMD* and the intergenic region between *LSM8* and *ANKRD7*, respectively—and was fully concordant with short‐read WGS (Figure 5). At the junctions, we detected additional structural complexity: a 12‐bp tandem duplication (chrX:32,488,589–32,488,600) immediately downstream of the X‐chromosome breakpoint and a 42‐bp deletion (chr7:118,206,978–118,207,019) upstream of the Chromosome 7 breakpoint.

**Figure 5:**
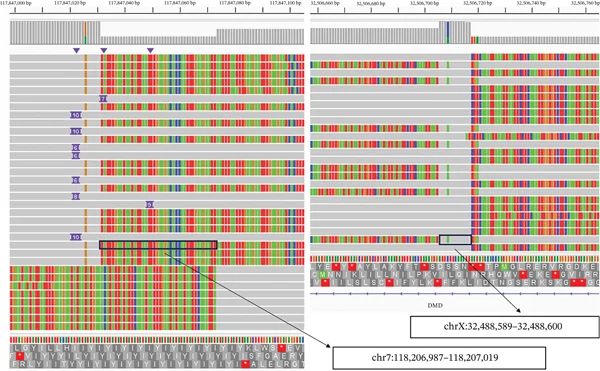
Long‐read WGS confirms the t(X;7) translocation junction with single‐nucleotide resolution (GRCh38). The alignment shows the precise breakpoint and associated structural complexity, including a 12‐bp tandem duplication on chrX and a 42‐bp deletion on chr7.

All SV coordinates identified by short‐read and long‐read WGS were converted from GRCh37/hg19 to GRCh38/hg38 using CrossMap v0.6.2 to enable direct comparison with OGM results and consistent annotation.

### 3.8. Karyotyping Confirms Inherited Reciprocal Translocation t(X;7)

Chromosome karyotype analysis confirmed a reciprocal translocation in the proband, showing 46 chromosomes with an exchange between the distal portions of Xp and 7q. Specific breakpoints could not be clearly identified due to limitations in karyotype resolution (Figure 6a). The mother′s karyotype similarly showed 46 chromosomes with the identical translocation (Figure 6b), indicating that the translocation was inherited from her.

Figure 6Peripheral blood karyotypes of the proband and his mother. Karyotype analysis of peripheral blood from the proband and his mother revealed subtle banding abnormalities at the distal ends of Xp and 7q, confirming the presence of the translocation.

**Figure:**
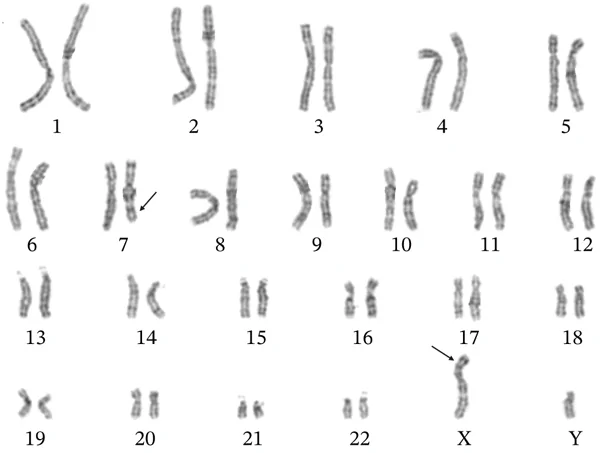
(a)

**Figure:**
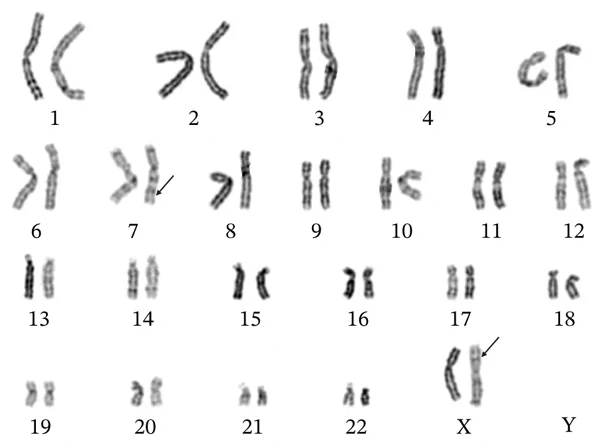
(b)

## 4. Discussion

The link between X;autosome translocations and dystrophinopathy in females was established through affected carriers with balanced translocations disrupting Xp21, providing early evidence for the *DMD* gene locus [7]. To date, over 40 such cases have been reported, involving 15 different autosomes—most frequently Chromosomes 9, 22, and 4—with no significant clustering, suggesting no strong autosomal breakpoint preference [8, 9]. Among these, the majority of carriers with X;autosome translocations involving Xp21 develop clinical symptoms, with a phenotypic spectrum ranging from asymptomatic to severe DMD‐like presentations, largely influenced by skewed X‐chromosome inactivation (XCI) [10–13]. Here, we report a rare male patient with a DMD‐like phenotype due to an inherited balanced X;autosome translocation, which is also present in his asymptomatic mother who exhibits a mild BMD‐like presentation, demonstrating that such rearrangements can result in dystrophinopathy in males without necessarily causing a more severe phenotype.

Several factors may explain the rarity of previously reported male cases. First, female carriers of X;autosome translocations frequently exhibit reproductive abnormalities—such as streak ovaries, infantile uterus, or premature ovarian insufficiency—leading to subfertility and recurrent pregnancy loss [14], which may reduce the likelihood of transmitting the translocation to offspring. Second, such translocations may be embryonically lethal in males due to the complete loss of functional dystrophin *and* the disruption of developmentally critical autosomal genes, a scenario not mitigated by XCI as in females [15]. Third, routine diagnostic methods for dystrophinopathy—such as Sanger sequencing and MLPA—are optimized for detecting intragenic mutations and copy number variations but often fail to identify balanced translocations [16]. Fourth, symptomatic female carriers are more likely to undergo early diagnosis, prompting genetic counseling and reproductive interventions that may prevent the birth of affected children [17]. In contrast, our patient survived into childhood with a clinically evident phenotype, likely due to a combination of a permissive breakpoint configuration, partial preservation of *DMD* function, and a nonlethal autosomal disruption inherited from his mildly affected mother.

Regarding the mechanism of translocation formation, studies suggest that specific sequence motifs, repetitive elements, and DNA structural features—such as fragile sites or non‐B DNA conformations—may mediate double‐strand breaks and illegitimate repair between nonhomologous chromosomes [18]. Additionally, higher order chromatin architecture, including topologically associating domains (TADs), may facilitate spatial proximity‐driven rearrangements [19]. The molecular characterization of such rearrangements has evolved from cytogenetic and Southern blot‐based methods to high‐resolution genomic technologies capable of mapping SVs with near‐nucleotide precision [7, 20–25]. In our case, an integrative approach combining OGM, short‐read WGS, and karyotyping enabled the detection and localization of a cryptic X;7 translocation. Long‐read WGS further revealed microduplications and deletions at the translocation junctions, features characteristic of error‐prone nonhomologous end joining (NHEJ) repair [26, 27]. To our knowledge, this is a rare balanced translocation causing dystrophinopathy to be resolved at single‐nucleotide resolution using long‐read WGS. However, the exact breakpoint could not be resolved by Sanger sequencing, likely due to structurally complex DNA at the junction. Furthermore, although RNA‐seq suggested potential aberrant splicing, this was not confirmed by RT‐PCR, highlighting the challenges in interpreting transcriptomic predictions for complex SVs.

## 5. Conclusion

We report a rare male patient with dystrophinopathy caused by a maternally inherited balanced reciprocal translocation disrupting *DMD* at Xp21, expanding the phenotypic spectrum of X;autosome rearrangements beyond symptomatic female carriers. This case highlights a persistent diagnostic gap: Despite negative standard testing, balanced translocations remain a hidden cause of *DMD* disruption in mutation‐negative males. Limitations in familial segregation analysis include the inability to exclude gonadal mosaicism in the mother and the unavailability of genetic data from the asymptomatic older brother. Our findings support the inclusion of genome‐wide SV analysis in the diagnostic workup of unresolved dystrophinopathies.

## Author Contributions

G.L. and K.Z. conceived and designed this study. Experiments were conducted by M.G. Data were analyzed by R.D. Clinical diagnosis of the subject was undertaken by K.M. The paper was written by R.D. and X.W. R.D. and K.M. contributed equally to this work.

## Funding

This study was supported by Science and Technology Development Program of Jinan Municipal Health Commission (2024304029).

## Conflicts of Interest

The authors declare no conflicts of interest.

## Supporting information


**Supporting Information** Additional supporting information can be found online in the Supporting Information section. Figure S1.

## Data Availability

The data that support the findings of this study are available on request from the corresponding authors. The data are not publicly available due to privacy restrictions.
